# Stachydrine Showing Metabolic Changes in Mice Exposed to House Dust Mites Ameliorates Allergen-Induced Inflammation

**DOI:** 10.3390/nu17122015

**Published:** 2025-06-16

**Authors:** Ji-Hye Do, Jung Yeon Hong, Ji-Hye Jang, Kyu-Tae Jeong, Seung Hyun Kim, Hye-Ja Lee

**Affiliations:** 1 Division of Allergy and Chronic Respiratory Diseases, Department of Chronic Disease Convergence Research, Korea National Institute of Health, Korea Disease Control and Prevention Agency, Osong 28159, Republic of Korea; doji0416@korea.kr (J.-H.D.); fogjkt@korea.kr (K.-T.J.); 2 Infectious Disease Inspection Division, Seoul Regional Office, Animal and Plant Quarantine Agency, Cheongju 07670, Republic of Korea; jikong0214@korea.kr; 3 Division of Regenerative Medicine Safety Control, Department of Chronic Disease Convergence Research, Korea National Institute of Health, Korea Disease Control and Prevention Agency, Cheongju 28159, Republic of Korea

**Keywords:** chronic airway inflammation, house dust mite, stachydrine

## Abstract

**Background/Objectives:** Asthma, a chronic airway inflammatory disease characterized by bronchial narrowing and caused by an inflammatory response, results in airway obstruction and hyperresponsiveness. Stachydrine (STA), an abundant metabolite found in plants and humans, is recognized for its bioactivity in treating fibrosis, cancer, and inflammation. However, its effects on asthma have not been fully elucidated. We aimed to investigate the ameliorating effects of STA on chronic airway inflammation caused by *Dermatophagoides pteronyssinus* (house dust mite, HDM). **Methods**: We used a murine model of HDM-induced airway inflammation to assess the change in metabolite profile by chronic airway inflammation. The mice were challenged with HDM (35 challenges in total) for up to 12 weeks. Serum metabolites were analyzed using capillary electrophoresis time-of-flight mass spectrometry. **Results**: HDM exposure increased airway hypersensitivity, immune cell infiltration, cytokine production, goblet cell hyperplasia, collagen deposition, and alpha smooth muscle actin and fibronectin expression. Serum metabolite analysis revealed that STA levels were lower in the mice with HDM-induced chronic inflammation than in the controls. In vitro analyses demonstrated that HDM sensitization increased cytokine production (interleukin [IL]-6 and IL-8) and extracellular signal-regulated kinase (ERK) activity. However, STA treatment reduced HDM-induced IL-6 and IL-8 production and ERK activity. Co-treatment with a mitogen-activated protein kinase (MAPK) inhibitor and STA resulted in a more pronounced reduction in cytokine production and MAPK activity. **Conclusions**: These findings suggest that STA, particularly when used in combination with a MAPK inhibitor, effectively suppresses airway inflammation through ERK pathway inhibition, making it a potential therapeutic agent for asthma treatment.

## 1. Introduction

Allergic asthma is a highly heterogeneous chronic inflammatory disease of the airways characterized by airway hyperresponsiveness (AHR), airflow obstruction, mucus hypersecretion, and airway remodeling. These processes are partly driven by eosinophilia, neutrophilia, and T cell activation [1,2]. Eosinophilic inflammation is the crucial aspect of asthma pathogenesis. Furthermore, asthma comprises distinct endotypes with different pathophysiological mechanisms, leading to complex clinical phenotypes [3]. Asthma phenotypes include eosinophilic, neutrophilic, and mixed-granulocytic asthma. Notably, neutrophil-dominant airway inflammation is often associated with persistent asthma, and patients with neutrophilia respond poorly to corticosteroids [4,5]. Therefore, alternative approaches are required to treat patients with uncontrolled asthma.

Metabolomics enables the detection of metabolites for phenotype categorization, specific biomarkers of human diseases, and certain changes after genetic and environmental interventions [6]. Metabolites, the intermediate or end products of cellular metabolism, are essential for maintaining biological homeostasis and normal cell function [7]. Therefore, alterations in metabolites can more accurately reflect physiological or pathological changes. Recent studies have revealed altered metabolic pathways associated with asthma pathogenesis [6,8]. However, the specific metabolites that may serve as key determinants of chronic airway inflammation remain unclear.

Stachydrine (STA), also known as proline betaine and N,N-dimethyl-L-proline, is a major component found in *Leonurus heterophyllus* Sweet, *L. japonicus*, *L. cardiaca* fruits, *Leonotis leonurus*, and citrus fruits. It exhibits several pharmacological properties, including antioxidant, anti-fibrotic, anticancer, and anti-inflammatory effects [9,10,11]. Recent studies have demonstrated that STA protects against carbon tetrachloride (CCl_4_)-induced hepatic fibrosis by inhibiting the expression of inflammatory factors such as interleukin (IL)-6, IL-8, COX-2, and IL-1β [12]. Additionally, it protects against transverse aortic constriction-induced myocardial hypertrophy and cardiac dysfunction by inhibiting cardiac fibrosis [13]. Moreover, STA was identified as a differential metabolite with decreased levels observed in the lungs and respiratory muscles of a ventilator-induced lung injury model [14]. Thus, we hypothesized that STA may be a novel candidate agent for treating chronic asthma by inducing the targeted suppression of airway inflammation and remodeling. However, the effects of STA on airway inflammation have not been investigated.

Therefore, we aimed to investigate whether STA is associated with the development of HDM-induced chronic airway inflammation and evaluated its therapeutic potential in an asthmatic mouse model. Moreover, we explored the effects of STA on HDM-induced inflammation in human airway epithelial cells.

## 2. Materials and Methods

### 2.1. Animals

Female BALB/c mice (6 weeks old) were purchased from DooYeol Biotech (Seoul, Republic of Korea). The mice were housed in a disposable individually ventilated cage system (Innovive, San Diego, CA, USA) under a 12 h light–dark cycle (lights on at 7 am) at the Animal Research Center of the Korea Centers for Disease Control and Prevention (KCDC). The animal care and laboratory protocols were approved by the Institutional Animal Care and Use Committee of the KCDC (KCDC-IACDC-02-040).

### 2.2. Chronic HDM-Induced Airway Inflammation Mouse Model

Chronic allergen-induced airway inflammation was induced in the mice using house dust mite (HDM) extract (*Dermatophagoides pteronyssinus*; Greer, Lenoir, NC, USA) solubilized in sterile phosphate-buffered saline (PBS). The mice received intranasal PBS (control group) or 25 µg HDM (HDM group) 35 times, three times a week for up to 12 weeks. Subsets of mice from each group (4–8 mice) were evaluated after 14 (PBS 5 mice, HDM 5 mice), 26 (PBS 6 mice, HDM 5 mice), and 35 (PBS 8 mice, HDM 4 mice) challenges and all samples (BALF, lung, and serum) were obtained the following day at each of the three time points.

### 2.3. AHR Measurement

AHR was measured using an invasive measurement system (flexiVent Fx1; SCIREQ, Montreal, QC, Canada) according to the manufacturer’s protocol. Regular ventilation and deep inflation measurements were conducted following exposure to different methacholine concentrations (0, 12.5, 25, and 50 mg/mL) (Sigma-Aldrich, St. Louis, MO, USA), as described previously [10].

### 2.4. Bronchoalveolar Lavage Fluid (BALF) Collection and Immune Cell Analysis

To collect BALF, the trachea was punctured with a syringe, and the lungs were flushed three times with 0.8 mL ice-cold PBS. The BALF was then centrifuged to pellet the cells, and red blood cells were removed using RBC Lysis buffer (Sigma-Aldrich, St. Louis, MO, USA). For leukocyte counts, the cells were attached to slides using Cytospin (Cellspin, Hanil, Kimpo, Republic of Korea) and stained with Diff-Quick solution (Sysmex Corporation, Hyogo, Japan).

### 2.5. Serum IgE and Cytokine Measurement

Peripheral blood was collected from the retro-orbital plexus and centrifuged to measure total and HDM-specific IgE levels. Total serum IgE levels were measured using an ELISA kit (Invitrogen, Vienna, Austria), according to the manufacturer’s protocol. To detect HDM-specific IgE, HDM extract (10 μg) was coated in each well of a 96-well plate, and the same procedure used for total IgE measurement was followed. Lung tissue was homogenized and centrifuged, and the supernatant was collected. IL-4, IL-5, IL-13, IL-17, IL-1 β, and TNF-α levels were measured in the lung tissue supernatant. Human bronchial epithelial cell (BEAS-2B; ATCC, Manassas, VA, USA) supernatants were collected and the levels of IL-6 and IL-8 were measured using an ELISA kit (R&D Systems, Inc., Minneapolis, MN, USA).

### 2.6. Histological Analysis of Lung Tissue

The left lung of each mouse was excised, fixed in paraformaldehyde, and embedded in paraffin. Tissue sections were stained with hematoxylin and eosin (H&E), PAS, and Sirius Red. Microscopic evaluation was conducted and the PAS- and Sirius Red-stained areas were digitized and measured using Image-Pro by Media Cybernetics, Inc. (Rockwille, MD, USA), version 10.10.

### 2.7. Metabolite Analysis

The serum was pooled into three samples per group for metabolite analysis. Metabolic analysis was performed using the basic scan package from Human Metabolome Technologies (HMT) and capillary electrophoresis time-of-flight mass spectrometry (CE-TOF-MS). Raw data peaks from CE-TOF-MS were analyzed using MasterHands automatic integration software, version 2.19.0.2 (Keio University, Tsuruoka, Yamagata, Japan) as previously described [2]. The peaks were annotated according to the HMT metabolite database, based on *m*/*z* values and migration time. The area under each annotated peak was normalized to the internal standard and sample amount to obtain the relative levels of each metabolite.

### 2.8. Cell Culture and HDM Exposure

BEAS-2B cells were cultured in bronchial epithelial cell growth medium (BEGM; BulletKit, Lonza) in a humidified atmosphere of 5% CO_2_ at 37 °C. Cells were seeded in 96-well plates (1 × 10^4^ cell/well) in BEGM. Once the cells reached confluence, they were pretreated with or without STA (10 μM or 25 μM for 4 h) or the extracellular signal-regulated kinase (ERK)1/2 selective inhibitor, U0126 (2.5 µM, for 1 h; Cell Signaling Technology, Danvers, MA, USA) in BEBM. Additionally, the cells were co-treated with 25 µM STA and 2.5 µM U0126. Following pretreatment, HDM extract (50 µg/mL) was added to the culture medium, and the cells were incubated for 48 h.

### 2.9. Western Blot Analysis

Mouse lung tissues were homogenized using a probe sonicator (VCX-130, Sonic and Materials, Newtown, CT, USA) in PRO-PREP Protein Extraction Solution (iNtRON Biotechnology, Sungnam, Republic of Korea) to lyse the cells and extract proteins, according to the manufacturer’s instructions. Proteins were separated using NuPAGE 4–12% Bis-Tris Protein Gels (Invitrogen, Carlsbad, CA, USA) and transferred onto polyvinylidene fluoride membranes. After blocking nonspecific binding sites, the blots were incubated overnight at 4 °C with primary antibodies specific to alpha smooth muscle actin (α-SMA), fibronectin (Abcam, Cambridge, UK), phospho (p)-p38, p38, p-c-Jun N-terminal kinase (JNK), JNK, p-ERK, ERK, glyceraldehyde 3-phosphate dehydrogenase (GAPDH), and β-actin (Cell Signaling Technology). Following incubation with horseradish peroxidase-conjugated secondary antibodies, the bands were visualized using enhanced chemiluminescence (ECL, Thermo Fisher Scientific, Waltham, MA, USA).

### 2.10. Statistical Analysis

Data are presented as the means ± standard error of the mean (SEM). Differences between groups were evaluated using one-way analysis of variance (ANOVA) and Tukey’s or the Mann–Whitney U test. Two-way ANOVA was also performed using Bonferroni post-tests to compare replicate means by row. All statistical analyses were performed using GraphPad Prism 5 (GraphPad Software, San Diego, CA, USA). Statistical significance was set at *p* < 0.05.

## 3. Results

### 3.1. HDM Challenge Induces Chronic Airway Inflammation

**Figure 1:**
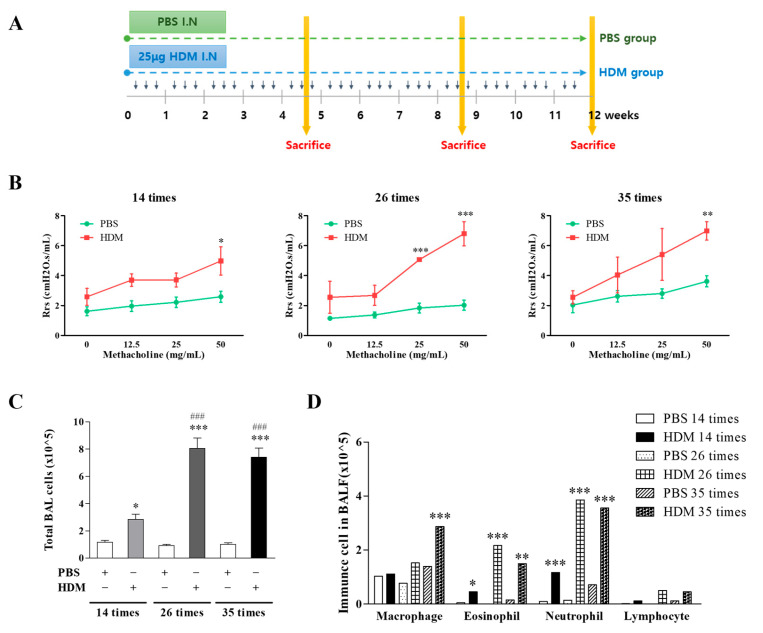
Protocol for chronic allergen-induced airway inflammation in a mouse model and outcomes of the HDM challenge. (**A**) Mice were intranasally challenged with PBS (control) or 25 µg HDM extract three times a week for 12 weeks (a total of 35 challenges, small arrows). AHR was assessed after 14, 26, and 35 challenges, and blood, BALF, and lung tissue were analyzed. (**B**) Methacholine responsiveness was measured after administering increasing doses of methacholine (0, 12.5, 25, and 50 mg/mL). (**C**,**D**) Total cell count and infiltration of neutrophils, eosinophils, and macrophages were measured in the BALF. HDM (* *p* < 0.05, ** *p* < 0.01, *** *p* < 0.001) vs. time-matched PBS (### *p* < 0.001). HDM, house dust mite; PBS, phosphate-buffered saline; AHR, airway hyperresponsiveness; BALF, bronchoalveolar lavage fluid.

We assessed AHR, cellular inflammation, and histological changes in a mouse model of HDM-induced chronic asthma (Figure 1A). As expected, the asthmatic mice exhibited a significant increase in lung resistance in response to methacholine exposure compared with the control mice (Figure 1B). Specifically, a significant increase in lung resistance was observed in the HDM group compared with the PBS control group across all time points when exposed to 50 mg/mL methacholine (14 challenges: 2.59 ± 0.64 vs. 4.98 ± 1.63, PBS vs. HDM, *p* < 0.05; 26 challenges: 2.02 ± 0.75 vs. 6.80 ± 1.39, *p* < 0.001; 35 challenges: 3.62 ± 0.90 vs. 6.99 ± 1.23, *p* < 0.01). Additionally, lung resistance significantly increased after 26 challenges when exposed to 25 mg/mL methacholine (1.83 ± 0.73 vs. 5.07 ± 0.20 cmH2O.s/mL, PBS vs. HDM, *p* < 0.001). The HDM-challenged mice showed an increase in total BALF cell counts compared with control mice. Among these groups, the 26-challenge HDM model exhibited the most significant cellular recruitment to the airways (Figure 1C; 0.92 ± 0.18 vs. 8.06 ± 1.49 × 10^5^, PBS vs. HDM, *p* < 0.001).

**Figure 2:**
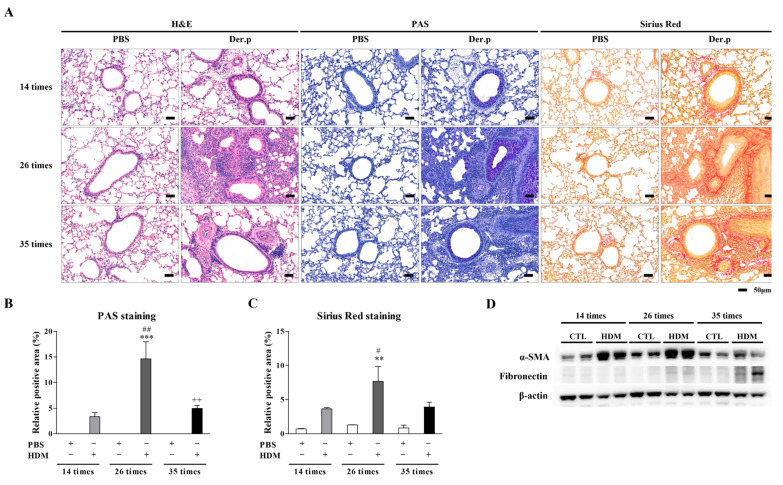
Airway remodeling assessed through histological analysis of lung tissue. (**A**) Lung sections were stained to evaluate inflammatory cell recruitment (H&E), goblet cell hyperplasia (PAS), and collagen deposition (Sirius Red) over time. Scale bar: 50 μm. (**B**,**C**) Staining degree was quantified as the ratio of the stained area to the total area challenges (** *p* < 0.01, *** *p* < 0.001) vs. time-matched PBS (# *p* < 0.05, ## *p* < 0.01) vs. 26 HDM challenges (++ *p* < 0.01). (**D**) Expression levels of α-SMA and fibronectin in whole lung tissue were determined by immunoblotting. 14 HDM. H&E, hematoxylin and eosin; PAS, periodic acid–Schiff; α-SMA, alpha smooth muscle actin; HDM, house dust mite; PBS, phosphate-buffered saline; CTL, control.

The absolute cell counts of macrophages, neutrophils, and eosinophils were higher in the BALF of the HDM-exposed mice than in that of the control mice (Figure 1D). Specifically, the eosinophil counts were significantly increased after 14, 26, and 35 challenges (*p* < 0.05, *p* < 0.001, and *p* < 0.01, respectively) compared with those in the control group. Similarly, the neutrophil counts were significantly higher after 14, 26, and 35 challenges (*p* < 0.001, *p* < 0.05, and *p* < 0.001, respectively). The macrophage counts were significantly increased in the BALF of the HDM-exposed mice after 35 challenges (*p* < 0.001) compared with those of the control mice. Histological analysis of the pulmonary lesions showed that the asthmatic mice exhibited pronounced perivascular and peribronchial cell infiltration and epithelial cell hyperplasia. Inflammatory cell infiltration, goblet cell hyperplasia, and collagen deposition were the most severe, particularly in the 26-challenge model, with some amelioration observed in the 35-challenge model (Figure 2A–C). Additionally, the expression of fibrosis-related factors, α-SMA and fibronectin, was significantly increased in the lungs of the HDM-exposed mice compared with that of the PBS controls. Fibronectin levels were increased in the 26- and 35-challenge HDM model mice compared with the controls (Figure 2D). These results suggest that chronic nasal exposure to HDM increases airway inflammation and fibrosis.

### 3.2. Inflammatory Mediator Expression

**Figure 3:**
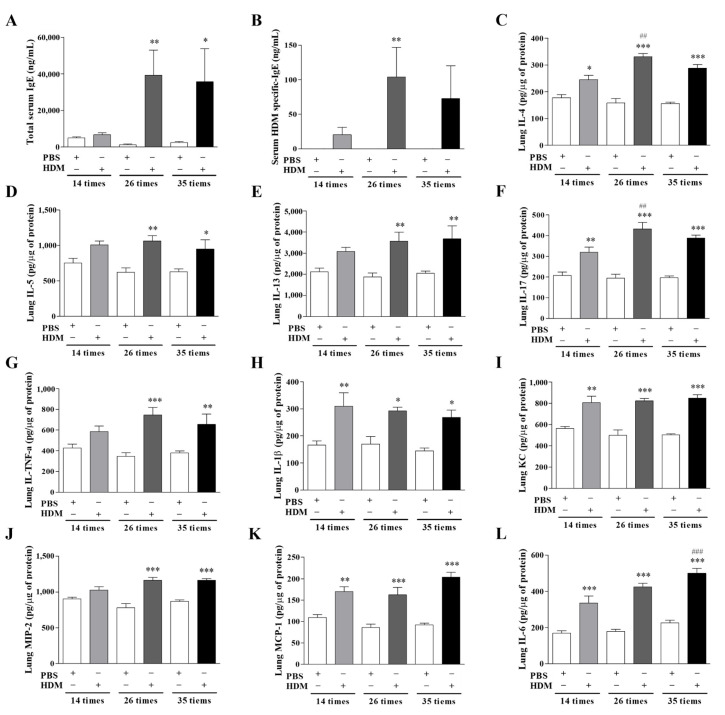
The levels of immunoglobulins and cytokines in mouse serum. (**A**) The total IgE and (**B**) HDM-specific IgE levels in mouse serum. (**C**–**L**) Levels of Th2 cell-type cytokines (IL-4, IL-5, IL-13), Th17-type cytokine (IL-17), inflammatory cytokines (IL-1β, TNF-α, IL-6), and chemokines (KC, MIP-2, MCP-1) were measured by ELISA. 14 HDM challenges (* *p* < 0.05, ** *p* < 0.01, *** *p* < 0.001) vs. time-matched PBS (## *p* < 0.01, ### *p* < 0.001). IgE, immunoglobulin E HDM, house dust mite; Th2, type 2 T helper; IL, interleukin; TNF-α, tumor necrosis factor alpha; MIP-2, macrophage inflammatory protein 2; MCP-1, monocyte chemoattractant protein 1; KC, keratinocyte-derived cytokine; ELISA, enzyme-linked immunosorbent assay (ELISA); HDM, house dust mite; PBS, phosphate-buffered saline.

To confirm the development of the allergic response, we investigated the total and HDM-specific immunoglobulin E (IgE) levels in the mouse blood serum (Figure 3A,B). The serum levels of total IgE were significantly elevated in the 26- and 35-challenge models (*p* < 0.01 and *p* < 0.05, respectively) than in the PBS controls (Figure 3). Additionally, the HDM-specific IgE levels were significantly elevated in the 26-challenge models compared with the controls (*p* < 0.01). We then quantified lung cytokines and observed significantly increased concentrations of IL-4, IL-5, IL-13, tumor necrosis factor alpha (TNF-α), IL-17, IL-1β, and IL-6 in the HDM-challenged mice than in the control mice. Specifically, the IL-4 levels were significantly elevated after 14, 26, and 35 challenges (*p* < 0.05, *p* < 0.001, and *p* < 0.001, respectively). The IL-5 levels were significantly increased after 26 and 35 challenges (*p* < 0.01 and *p* < 0.05, respectively). Similarly, the IL-13 (*p* < 0.01 for both challenges) and TNF-α levels (*p* < 0.001 and *p* < 0.01, respectively) were significantly increased after 26 and 35 challenges. The IL-17 levels were significantly higher in the HDM-exposed mice after 14, 26, and 35 challenges (*p* < 0.05, *p* < 0.001, and *p* < 0.001, respectively) than in the control mice. Moreover, IL-1β (*p* < 0.01, *p* < 0.05, *p* < 0.05, respectively) and IL-6 (*p* < 0.001, *p* < 0.001, *p* < 0.001, respectively) were significantly increased in the asthmatic mice after 14, 26, and 35 challenges. The neutrophil chemokine keratinocyte-derived cytokine (KC) (CXCL1) and macrophage inflammatory protein 2 (MIP-2, CXCL2), which play an important role in the migration and infiltration of monocytes and macrophages, were also significantly increased after 14, 26, and 35 challenges. (Figure 3C–L).

### 3.3. Metabolite Profile Analysis in Mouse Serum

To identify metabolite profiles, we assessed the serum metabolites in the mice chronically exposed to HDM. In the 14-challenge group, the levels of 20 metabolites increased, whereas those of 6 metabolites decreased compared with those in the PBS group. The increased metabolites were associated with pathways such as amino sugar and nucleotide sugar metabolism, carbon fixation in photosynthetic organisms, galactose and fructose metabolism, purine and pyrimidine metabolism, pyruvate metabolism, and Trp and Tyr metabolism. In the 26-challenge group, the levels of all the endogenous metabolites decreased. These metabolites were associated with the pathways related to glucosinolate biosynthesis, glycerolipid metabolism, Met and Phe metabolism, protein digestion and absorption, and Val, Leu, and Ile metabolism. In the 35-challenge group, the levels of four metabolites increased, whereas those of three metabolites decreased. The altered pathways included butanoate metabolism, protein digestion and absorption, and methane metabolism. Among the key metabolites, 2-aminoisobutyric acid/2-aminobutyric acid, a metabolite of the microbiome, significantly increased after 14 challenges but significantly decreased after 26 challenges. Also, STA, an NF-kB inhibitor, showed the most significant changes in line with HDM-specific IgE after long-term challenges with HDM, suggesting its potential role in modulating inflammation during chronic allergen exposure. Thus, we performed further functional studies using in vitro cell model.

### 3.4. Effect of STA on HDM-Induced Inflammation in BEAS-2B Cells

**Figure 4:**
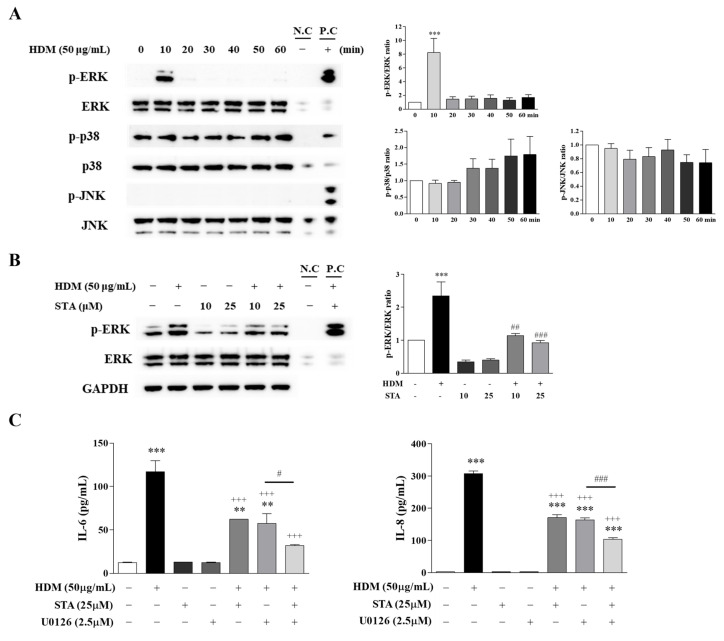
Effect of STA on human bronchial epithelial cells (BEAS-2B). (**A**) Phosphorylation levels of ERK, p38, and JNK in cells treated with HDM. A time-course analysis was conducted at 10 min intervals for up to 60 min. (**B**) Cells were pretreated with STA (10 or 25 μM) for 4 h, followed by treatment with 50 μg/mL HDM for 10 min. The fold change was measured as the phosphorylation-to-total ratio of ERK. (**C**) Cells were pretreated with STA for 4 h and U0126 for 1 h, followed by 48 h HDM exposure. No pretreatment (** *p* < 0.01, *** *p* < 0.001) vs. PBS control (+++ *p* < 0.001) vs. HDM + U0126 (# *p* < 0.05, ## *p* < 0.01, ### *p* < 0.001). STA, stachydrine; ERK, extracellular signal-regulated kinase; JNK, c-Jun N-terminal kinase; HDM, house dust mite; p-, phospho; U0126, an ERK1/2 inhibitor; PBS, phosphate-buffered saline; GAPDH, glyceraldehyde 3-phosphate dehydrogenase; N.C, normal control; P.C, positive control.

The airway epithelium provides the first defense barrier against the environment and collaborates with resident and recruited immune cells to regulate airway immunity [15]. However, cell barrier dysfunction is frequently observed in allergic diseases, which are linked to inflammatory cytokine and chemokine release processes [16]. In an in vitro setting, we exposed airway epithelial cells to HDM and subsequently assessed mitogen-activated protein kinase (MAPK) activation by performing western blot analysis. A time-course analysis (0, 10, 20, 30, 40, 50, and 60 min) showed that HDM challenge induced rapid phosphorylation of ERK, peaking within 10 min, whereas the phosphorylation of p38 MAPK and JNK did not change through various time points (Figure 4A).

We observed significantly reduced serum STA concentrations in the mice challenged with HDM 26 times than in the serum of the PBS control mice (Table 1). Therefore, we explored the effect of STA on HDM-induced BEAS-2B cell inflammation to determine its anti-allergic action. To determine whether ERK phosphorylation influences HDM-induced epithelial cell inflammation, STA was used to downregulate ERK activation. Pretreatment with STA decreased HDM-induced ERK phosphorylation (2.35 ± 0.74 vs. 1.14 ± 0.11, no pretreatment vs. 10 µM STA pretreatment, *p* < 0.01; 2.35 ± 0.74 vs. 0.92 ± 0.13 ratio, no pretreatment vs. 25 µM STA pretreatment, *p* < 0.001; Figure 4B). We then investigated whether the ERK pathway was related to the HDM-stimulated production of proinflammatory cytokines. HDM-induced IL-6 and IL-8 production was significantly decreased by STA pretreatment (IL-6: 116.90 ± 26.02 vs. 62.24 ± 0.07, no pretreatment vs. 25 µM STA pretreatment, *p* < 0.001; IL-8: 307.14 ± 15.80 vs. 170.95 ± 17.90, *p* < 0.001; Figure 4C). Furthermore, the combination treatment with STA and the MAPK/ERK kinase (MEK) inhibitor U0126 significantly inhibited HDM-induced IL-6 and IL-8 production compared with treatment with STA or U0126 alone (IL-6: 57.33 ± 16.09 vs. 31.80 ± 2.06, U0126 alone vs. STA + U0126, *p* < 0.05; IL-8: 163.62 ± 11.89 vs. 102.80 ± 9.11 pg/mL, *p* < 0.001; Figure 4C). These results suggest that STA exerts a protective role against HDM-induced airway epithelial inflammation by inhibiting ERK phosphorylation and reducing the production of proinflammatory cytokines (IL-6 and IL-8). Additionally, combining STA with a MEK inhibitor enhances its anti-inflammatory effects.

## 4. Discussion

Various immune cell types contribute to asthma pathology [1]. Predominant neutrophilic inflammation has been associated with steroid resistance in individuals with stable asthma. Additionally, neutrophil recruitment is a significant characteristic of acute exacerbations in chronic asthma [5]. However, the specific phenotype of neutrophil-driven inflammation in chronic airway inflammation remains underexplored. In this study, we provide insights into the role of metabolic profiles and the potential of STA as a novel therapeutic agent for allergic asthma.

Persistent allergen exposure or inflammation can evolve into chronic allergic inflammation, which is characterized by the infiltration of various immune cells from both the innate and adaptive systems [1,2,17]. In respiratory diseases, such infiltration can eventually lead to respiratory structural remodeling and altered lung function. In this study, we successfully constructed a mouse model of chronic asthma, characterized by predominant infiltration of eosinophils, macrophages, T cells, and neutrophils, with the parallel development of AHR (Figure 1B–D). Analysis of the lung tissue revealed that mucus hyperplasia, airway thickness, and collagen deposition were significantly increased in the 26-challenge HDM group compared with those in the control group (Figure 2A–D). Notably, the 35-challenge HDM group showed reduced cell infiltration and epithelial thickening but exhibited increased size and thickening of blood vessels compared with the 26-challenge HDM group (Figure 2A–C). Moreover, we observed increased expression of SMA-α and fibronectin protein in the lung tissue and subepithelial fibrosis in chronic asthmatic airways (Figure 2A,D).

We observed significantly increased levels of type 2 T helper (Th2) cell cytokines (IL-4, IL-5, IL-13), neutrophil-related cytokines (KC, monocyte chemoattractant protein 1 [MCP-1], IL-17), proinflammatory cytokines (IL-1β, TNF-α), and profibrogenic mediators (TGF-β1), along with increased serum total and HDM-specific IgE levels (Figure 3). The classical asthma immune response is primarily driven by allergen-specific Th2 and type 2 innate lymphoid cells, which produce mainly IL-4, IL-5, and IL-13 [18]. Proinflammatory cytokines (TNF-α, IL-6) and profibrogenic mediators (TGF-β1, IL-13) also play significant and distinct roles in asthma pathogenesis. These cytokines are widely considered as the major effectors of airway inflammation [19], along with the involvement of other cell types, including epithelial cells of the mucosa, dendritic cells, mast cells, basophils, and eosinophils [18,20]. Th1 and Th17 cells also cooperate with the Th2 response to promote pathogenic inflammation [21]. In patients with asthma, IL-17 levels are increased in the airway and enhance fibroblast activity, leading to increased collagen deposition [22]. Additionally, Th17 can contribute to chronic inflammation associated with asthma and mediates steroid-resistant airway inflammation [5,23]. Moreover, elevated IL-6 levels, which promote Th2 activation and allergic responses while inhibiting regulatory T cell activity, are found in the airways of patients with asthma. TNF-α levels are increased in both adult and pediatric asthma patients, particularly those with corticosteroid-resistant asthma. Therefore, inhibiting or regulating these cytokines is a key strategy in asthma therapy [18,24].

Recent advancements in metabolomics have provided valuable insights into the metabolic changes associated with asthma [25]. Metabolic phenotypes of T effector cells in mild and severe asthmatics differ from those in healthy controls. Neutrophilic asthma, characterized by the involvement of both Th2 and Th17 subsets, is also associated with the increased expression of proteins with high metabolic activity [26,27]. Notably, these metabolic profiles can be pharmacologically targeted in vivo to mitigate airway inflammation and associated pathological features. A recent study demonstrated that aromatic amino acid (AAA) metabolites—tryptophan, phenylalanine, and tyrosine—have emerged as critical modulators of immune function. In particular, these amino acids and their metabolites are more than just blockers; they also play active roles in the intricate network of immunological responses, influencing cell function, inflammation, and immune homeostasis [28]. However, the role of metabolic phenotypes in regulating chronic airway inflammation remains poorly understood. In this study, we determined the metabolic profiles of an HDM-induced chronic asthma mouse model, differentiating between control and HDM exposure groups (14, 26, and 35 challenges). Of the 48 identified metabolites, none was significantly differentially regulated across the groups. Notably, all 15 endogenous metabolites significantly decreased after 26 HDM challenges (Table 1). Notably, STA was the only metabolite significantly associated with chronic HDM exposure (26 challenges). In vivo analyses revealed a strong negative correlation between serum STA concentration and neutrophil or eosinophil counts, lung function, and IgE levels in asthmatic mice (Figure 1, Figure 2 and Figure 3).

Given that asthma is a chronic condition, treatments such as adrenergic receptor (β_2_-AR) agonists and steroids are essential for disease management. Although these medications are effective, the long-term use of steroids in asthma treatment is restricted because of their significant side effects. Consequently, it is important to identify non-steroidal treatments that can be used for long durations without causing negative effects. STA has demonstrated various bioactivities for treating fibrosis [11], cardiovascular diseases [29], cancers [30,31], uterine diseases [32], brain injuries [33], and inflammation [33]. STA also exerts significant anti-fibrotic effects, and can moderate or reverse tissue fibrosis injury by reducing extracellular matrix deposition and decreasing inflammatory response [32]. In a rat model of cardiac fibrosis and hypertrophy, STA administration showed protective effects against oxidative stress and inflammation by inhibiting the ACE/AngII/AT1R-TGF-β1 axis [13]. Additionally, STA derived from *Pericarpium citri reticulatae* extract has shown potent anti-asthmatic and anti-tussive effects in a pig model of citric acid-induced cough [34]. Moreover, the combination of synephrine and STA demonstrated a stronger spasmolytic activity than either compound alone [35]. In this study also, the combined treatment with STA and U0126 exhibited an enhanced anti-inflammatory effect on HDM-induced cell inflammation. In a chronic mouse model, repeated allergen challenges led to an increase in inflammation and fibrosis, while STA levels decreased (Table 1). In this study, STA supplementation with an inhibitor reduced the mechanisms that induce asthma, suggesting that STA may have therapeutic potential, particularly for challenging conditions like neutrophil-dominant asthma.

Epithelial cells, as the first line of defense against environmental factors, are essential components of the innate immune response. Recent studies have highlighted the importance of metabolic regulation in maintaining mucosal barriers during allergic responses [36]. Here, we demonstrated the protective effect of STA in allergen-induced airway inflammation, consistent with its known anti-inflammatory and anti-fibrosis activities in various disease models. As expected, HDM exposure strongly stimulated ERK and proinflammatory cytokine release in human airway epithelial cells. We identified STA as an effective anti-inflammatory compound that significantly suppressed HDM-induced inflammatory pathways (Figure 4). Furthermore, the combination of an ERK inhibitor and STA significantly reduced the production of IL-6 and IL-8 in airway epithelial cells compared with either compound alone, indicating a potential synergistic effect. However, this study has limitations. There is a need for further in vivo evaluation of STA’s therapeutic potential at various dosages. In addition, high steroid resistance is a major problem in the treatment of neutrophil-dominant chronic airway inflammation, and it is necessary to confirm in vivo whether STA is a good therapeutic agent.

## 5. Conclusions

This study highlights the significant role of STA in modulating chronic airway inflammation. STA demonstrated moderate anti-inflammatory and anti-fibrotic effects via reducing ERK activation and proinflammatory cytokine release in airway epithelial cells. The combination of STA with an ERK inhibitor further enhanced these effects, suggesting its synergistic potential for asthma treatment. These findings provide promising evidence for STA as a non-steroidal therapeutic agent that could offer long-term benefits in managing asthma-related inflammation, particularly in steroid-resistant cases.

## Figures and Tables

**Table 1:** Metabolites changes in serum.

Compound Name	PubChem CID	14 PBS vs. 14 Der.p			26 PBS vs. 26 Der.p			35 PBS vs. 35 Der.p			Up/Down		
		Ratio	*p*-Value		Ratio	*p*-Value		Ratio	*p*-Value				
1-Methyladenosine	27476	1.2	0.034	*	1.0	0.503		1.2	0.446		↑	-	-
2-Hydroxybutyric acid	440864	1.6	0.015	*	0.8	0.156		1.1	0.515		↑	-	-
Dihydroxyacetone phosphate	668	1.6	0.016	*	0.7	0.424		0.9	0.483		↑	-	-
Fructose 6-phosphate	603	2.0	0.005	**	0.6	0.371		0.7	0.111		↑	-	-
Gluconic acid	10690	1.4	0.041	*	0.9	0.236		1.1	0.434		↑	-	-
Glucosamine 6-sulfuric acid	72361	2.2	0.024	*	<1	N.A.		N.A.	N.A.		↑	-	-
Glucose 1-phosphate	65533	1.7	0.025	*	0.6	N.A.		0.5	0.155		↑	-	-
Glucose 6-phosphate	5958	1.8	0.001	**	0.5	0.296		0.6	0.146		↑	-	-
N,*N*-Dimethylglycine	673	1.2	0.025	*	0.9	0.215		1.0	0.971		↑	-	-
Nicotinamide	936	1.3	0.012	*	0.6	0.157		1.0	0.676		↑	-	-
Ribose 5-phosphate	439167	1.8	0.017	*	0.6	0.280		0.8	0.588		↑	-	-
Ribulose 5-phosphate	439184	1.5	0.012	*	0.6	0.170		0.9	0.693		↑	-	-
S-Methylglutathione	115260	1.4	0.038	*	1.0	N.A.		0.9	0.546		↑	-	-
Sedoheptulose 7-phosphate	165007	2.1	0.013	*	0.7	0.539		0.7	0.261		↑	-	-
Serotonin	5202	1.2	0.010	**	1.0	0.851		0.7	0.266		↑	-	-
Succinic acid	1110	1.6	0.006	**	0.7	0.469		1.0	0.866		↑	-	-
Uracil	1174	1.8	0.031	*	0.6	0.131		0.8	0.211		↑	-	-
Xanthosine	64959	2.0	0.044	*	0.9	0.826		0.7	0.437		↑	-	-
Anserine_divalent	112072	0.7	0.038	*	0.8	0.293		1.2	0.505		↓	-	-
Citrulline	9750	0.8	0.002	**	0.9	0.214		1.1	0.507		↓	-	-
N-Acetylglycine	10972	0.8	0.011	*	0.9	0.365		1.1	0.622		↓	-	-
N-Acetyltryptophan	439917	0.6	0.040	*	0.6	0.089		1.1	0.774		↓	-	-
γ-Glu-Asn	131801686	0.8	0.005	**	0.9	0.538		1.1	0.236		↓	-	-
γ-Glu-Trp	3038501	0.7	0.048	*	0.9	0.305		1.0	0.519		↓	-	-
1-Methylhistidine 3-Methylhistidine	92105 64969	1.0	0.972		0.8	0.026	*	1.0	0.788		-	↓	-
3-Phosphoglyceric acid	439183	1.2	0.378		0.5	0.040	*	1.0	0.816		-	↓	-
Carnosine	439224	0.9	0.604		0.7	0.032	*	0.9	0.792		-	↓	-
Ile	791	1.0	0.913		0.8	0.018	*	1.0	0.848		-	↓	-
Leu	857	0.9	0.336		0.8	0.042	*	0.8	0.200		-	↓	-
Met	876	1.0	0.387		0.9	0.038	*	1.0	0.837		-	↓	-
N^6^,*N*^6^,*N*^6^-Trimethyllysine	440120	1.0	0.774		0.7	0.030	*	1.0	0.787		-	↓	-
SDMA	169148	1.1	0.108		0.7	0.015	*	1.0	0.847		-	↓	-
Tyr	1153	1.0	0.535		0.8	0.038	*	0.9	0.594		-	↓	-
XA0035		0.9	0.276		0.9	0.026	*	1.1	0.629		-	↓	-
XC0001		0.9	0.528		0.8	0.012	*	0.9	0.691		-	↓	-
XC0029 Stachydrine	0 115244	1.0	0.702		0.8	0.007	**	0.8	0.276		-	↓	-
γ-Glu-Arg_divalent	20719180	1.0	0.954		0.9	0.048	*	1.1	0.321		-	↓	-
γ-Glu-Ser	22844748	1.0	0.470		0.9	0.025	*	1.2	0.139		-	↓	-
Butyric acid Isobutyric acid	264 6590	1.4	0.229		1.0	0.983		1.3	0.022	*	-	-	↑
N-Acetylserine	65249	1.0	0.920		0.8	0.145		1.5	0.038	*	-	-	↑
XA0012		1.1	0.584		0.8	0.361		1.4	0.048	*	-	-	↑
XA0019		0.9	0.513		1.2	0.632		1.9	0.039	*	-	-	↑
Trimethylamine *N*-oxide	1145	1.3	0.289		0.9	0.243		0.7	0.049	*	-	-	↓
XC0040		1.3	0.289		0.9	0.243		0.7	0.049	*	-	-	↓
2-Aminoisobutyric acid 2-Aminobutyric acid	6119 6657	1.2	0.024	*	0.7	0.041	*	1.0	0.802		↑	↓	-
Ethyl glucuronide	18392195	1.3	0.039	*	N.A.	N.A.		0.6	0.037	*	↑	-	↓

* *p* < 0.05, ** *p* < 0.01. Arrows (↑; up and ↓; down) represent a increase or decrease in metabolite levels with significant changes in HDM-exposed mice after 14, 26, and 35 challenges compared with control mice. N.A.; not available.

## Data Availability

The original contributions presented in this study are included in the article. Further inquiries can be directed to the corresponding authors.

## Abbreviations

The following abbreviations are used in this manuscript:

STA	Stachydrine
HDM	House dust mite
IL	Interleukin
ERK	Extracellular signal-regulated kinase
